# Structural Motifs in Aryl Organogermanium Ge-O Derivatives for Material Design

**DOI:** 10.3390/ijms241713575

**Published:** 2023-09-01

**Authors:** Kirill V. Zaitsev, Igor S. Makarov, Yuri F. Oprunenko, Victor A. Tafeenko, Elmira Kh. Lermontova, Andrei V. Churakov

**Affiliations:** 1 Chemistry Department, M.V. Lomonosov Moscow State University, Leninskye Gory 1, 3, Moscow 119991, Russia; oprunenko@mail.ru (Y.F.O.); tafeenko-victor@yandex.ru (V.A.T.); 2 A.V. Topchiev Institute of Petrochemical Synthesis, Russian Academy of Sciences, Leninsky Pr. 29, Moscow 119991, Russia; makarov@ips.ac.ru; 3 N.S. Kurnakov General and Inorganic Chemistry Institute, Russian Academy of Sciences, Leninskii Pr. 31, Moscow 119991, Russia; elmira.ler@gmail.com (E.K.L.); churakov@igic.ras.ru (A.V.C.)

**Keywords:** group 14 elements, organogermanium compounds, germoxane, XRD analysis, organometallic materials

## Abstract

The aim of this work was to understand the main structural features and ways of formation of Ge-O bonds in organogermanium compounds under the conditions of Ar_n_GeHal_4-n_ (Hal = halide) hydrolysis. The structural types of these compounds were considered, providing 11 blocks (**A**–**K**). The molecular structures of the novel compounds [(*p*-FC_6_H_4_)_3_Ge]_2_O (**1**), [(*p*-F_3_CC_6_H_4_)_3_Ge]_2_O (**2**), and *cyclo*-[(*p*-F_3_CC_6_H_4_)_2_GeO]_4_ (**3**) were studied through XRD (X-ray diffraction) analysis. The molecular structure of [(*p*-F_3_CC_6_H_4_)_3_GeO]_4_Ge (**4**), representing a novel structural type, was also investigated. The data presented in this study will be important in the design of materials with useful properties based on group 14 element derivatives with element–oxygen bonding.

## 1. Introduction

Derivatives of group 14 elements (E = Si, Ge, Sn, and Pb) with E-O-E bonds are one of the most important classes of the main group compounds. Silicones [1,2] and organosilsesquioxanes, [RSiO_1.5_]_n_ [3,4], containing Si-O-Si bonds, attract significant attention from researchers due to their unique properties (including their chemical stability and low toxicity) and high potential in the field of material chemistry [5,6]. There are several structural types of these compounds, such as linear polymers, ladders, cages, and sheet-like structures. The Ge analogs of organosilsesquioxanes [7] and germanates [8,9,10] have attracted less attention. It is evident, however, that the introduction of germanium, instead of silicon, atoms (with a larger atom size and more acute bond angles) would significantly modify the structural features of compounds and their properties. Thus, the pentacyclic germasilsesquioxane cage may behave as a zeolite analog to form an inclusion compound with anions (usually with fluorides F^−^) [11,12], while other germanates may form complexes with metal cations [13], or provide ionic liquids [14], organo–inorganic frameworks [15], nanoporous materials [16], reagents in organic synthesis (in different cross-coupling reactions) [17,18], and biologically active substances [19]. Furthermore, compounds with Ge-O-Ge building blocks may behave as highly viscous biocompatible oils [20], ligands for transition metal ions [21], optical temperature [22] or organic vapor [23] sensors, Lewis acids, and catalysts. The problem underlying the directed synthesis of Ge-O-Ge-type compounds, and in establishing their properties and mechanisms of formation deserves significant attention. Introduction of a functionally substituted organic group to the Ge atom significantly changes the properties and structural features of the whole molecule.

The aim of this work was to synthesize several Ge-O-Ge derivatives with acceptor aryl groups (Ar = *p*-FC_6_H_4_ and *p*-F_3_CC_6_H_4_), investigate their structure through the employment of XRD (X-ray diffraction) analysis, and review the known Ge-O structures to understand their main features and principles of formation. Interestingly, the reactivity of organogermanium derivatives containing aromatic electron-withdrawing substituents has been scarcely studied to date, though their unusual chemical behavior has been demonstrated on several examples (e.g., the scission of Ge-C bonds in C_6_F_5_ derivatives using nucleophiles [24]). At the same time, the effect of organic electron-withdrawing substituents on the Ge atom in related compounds strongly affects the structure of the molecule (causing a change in conformation; as a rule, a decrease in the values of valent bonds and angles; packing in more ordered structures) [25], which makes it important to study the impact of electron-withdrawing substituents on the structure and properties of the Ge-O-Ge molecule. This article is a continuation of our research program [26,27,28] on the effect of substituents on group 14 element derivatives on their structures and properties.

## 2. Results

**Scheme 1:**
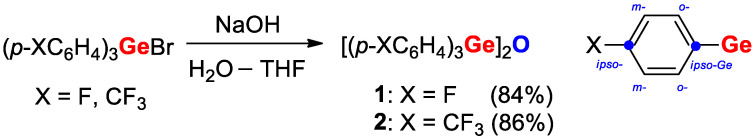
Synthesis of the germoxanes [(*p*-FC_6_H_4_)_3_Ge]_2_O (**1**) and [(*p*-F_3_CC_6_H_4_)_3_Ge]_2_O (**2**); conventions for carbon atoms in the Ar rings.

Compounds [(*p*-FC_6_H_4_)_3_Ge]_2_O (**1**) and [(*p*-F_3_CC_6_H_4_)_3_Ge]_2_O (**2**) were formed on the basis of Ar_3_GeHal (i.e., through hydrolysis of Ar_3_GeBr); the presence of these aromatic groups with substituents in the *p*-position strongly promotes crystallization to form monocrystals that are suitable for XRDanalysis. An effective way of germoxane synthesis consists of the action of aqueous NaOH on Ar_3_GeBr in THF (tetrahydrofuran) (Scheme 1); the hydroxide anion is a strong nucleophile for the Ge atom in the substitution of bromide. Water and THF were mixed, providing a homogeneous solution. Germoxane-type species, Ge-O-Ge, were subsequently formed due to the low stability of intermediate Ge-OH derivatives [29], which is typical of group 14 elements in the case of sterically unhindered substituents at the central atom. Among other known methods for the synthesis of R_3_Ge-O-GeR_3_ [29], the actions of aqueous NaOH [30] or Ag_2_CO_3_ [31] on R_3_GeHal is the simplest method. It should be noted that the high yields of the isolated products **1** and **2** (84–86%) indicate the high stability of Ge-C bonds in these Ar derivatives.

**Scheme 2:**
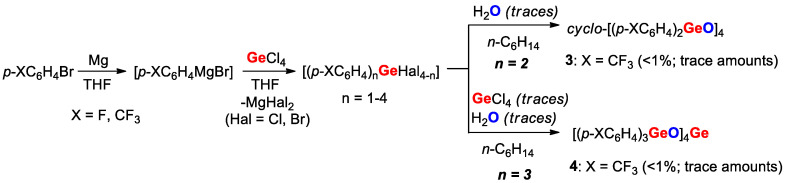
Synthesis of the germoxanes *cyclo*-[(*p*-F_3_CC_6_H_4_)_2_GeO]_4_ (**3**) and [(*p*-F_3_CC_6_H_4_)_3_GeO]_4_Ge (**4**).

Due to the high sensitivity of the Ge-Hal bond (Hal = Cl or Br), even to the trace of water, arylgermanium compounds are easily hydrolyzed, yielding different germoxanes, which may be observed at the isolation stage during the slow crystallization process. As stated earlier [32], the classical Grignard synthesis of arylgermanium halides via GeCl_4_/ArMgHal interactions (especially aimed at the synthesis of Ar_3_GeHal) usually yields an in situ complex mixture of differently substituted germanes, [(*p*-XC_6_H_4_)_n_GeHal_4-n_] (n varies within the range from one to four), due to the high reagent reactivity, high concentrations, and the orders and rates of reagent mixing. In this study, we found that the mother liquids, following the crystallization of the reaction mixtures in the synthesis of (*p*-XC_6_H_4_)_3_GeHal [32], yielded trace amounts of germoxanes (Scheme 2). Thus, the compounds *cyclo*-[(*p*-F_3_CC_6_H_4_)_2_GeO]_4_ (**3**) and [(*p*-F_3_CC_6_H_4_)_3_GeO]_4_Ge (**4**) were isolated; their molecular structures were studied through single crystal XRD analysis. It should be noted that the synthesis of *cyclo*-[(*p*-F_3_CC_6_H_4_)_2_GeO]_4_ (**3**) under hydrolysis of intermediate [(*p*-XC_6_H_4_)_2_GeHal_2_] (Hal = Cl or Br) represents an expected process. The formation of a specific compound strongly depends on the structure of the initial derivative (i.e., the steric size and the electronic properties of Ar or R) and, to a lesser extent, on the conditions used. Usually, cyclic [R_2_GeO]_n_ (n = 3 or 4) species are formed in diluted solutions following the hydrolysis of R_2_GeHal_2_ by NaOH/H_2_O in acetone [33] or in xylene [34], Et_3_N/H_2_O [23,35,36], or through the thermal actions of Ag_2_CO_3_ on R_2_GeHal_2_ [37]. The synthesis of *cyclo*-[Ph_2_GeO]_3_ was observed by Glockling et al. at a rare cleavage of (Cl_3_CCO_2_)Ph_2_Ge-GePh_2_(O_2_CCCl_3_) by NaOH/H_2_O [38]. Interestingly, analysis of the data available in the literature showed that the four-membered *cyclo*-[R_2_GeO]_2_ cycles were not formed under R_2_GeHal_2_ hydrolysis; other methods were used in this case, including the oxidation of the digermenes, R_2_Ge=GeR_2_ [39,40,41], or the germynes, RGe≡GeR [42]. 

The synthesis of the unusual [(*p*-F_3_CC_6_H_4_)_3_GeO]_4_Ge (**4**) was attributed to the interaction of intermediate [(*p*-XC_6_H_4_)_3_GeHal] (Hal = Cl or Br), formed in situ during Grignard synthesis, with GeCl_4_ present in the mother liquids with random water; here, we observed the tendency to form Ge-O-Ge species under equilibrium conditions. It should be emphasized that reactions of such a type, where a germoxane structural block forms from different Ge sources, are highly scarce. To the best of our knowledge, only Draeger et al. described the hydrolysis of PhGeCl_3_/Ph_2_GeCl_2_ (2:3) using aqueous AgNO_3_, forming *bicyclo*-[PhGe(Ph_2_GeO_2_)_3_GePh] [43].

Generally, [Ar_n_Ge(OH)_4−n_] compounds (n = 2 or 3) are the evident intermediates that are observed during the synthesis process in the hydrolysis reactions. The real existence of such molecules is only possible in the case of sterically huge groups (e.g., *t*-Bu_2_Ge(OH)_2_ studied with the aid of XRD analysis by Puff et al. [34]; [Fc(Ph)Ge(OH)]_2_O, by Pannell et al. [36]; [(*t*-Bu)Ge(OH)]_2_O, by Jurkschat et al. [44]; and [(Me_2_NCH_2_Fc)Ge(OH)O]_3_, by Lorenz et al. [45]) and, more intriguingly, for electron-withdrawing groups (e.g., (F_5_C_2_)_2_Ge(OH)_2_ [37] and (F_5_C_2_)_3_GeOH [31], investigated by Hoge et al.). As an obvious proof of this statement, the synthesis of compound **4** can be considered, where Ar_3_Ge-O-H species reacted with the Ge-Cl compound (GeCl_4_), formingg thermodynamically favorable Ge-O-Ge bonds. As emphasized by different researchers, including Draeger et al. [46], the hydrolytic condensation of molecular compounds (R_3_GeX; X = Hal, OR’, etc.) may result in the formation of cyclic trimers or tetramers or polymeric materials (with terminal a OH for R_2_GeX_2_), or in cage and polymer structures (for RGeX_3_ derivatives).

The composition and identity of compounds **1**–**4** was confirmed using mass spectrometry and elemental analysis data; for germoxanes **1** and **2**, their structures were also studied using multinuclear NMR spectroscopy (^1^H, ^13^C, and ^19^F; for details, see Supplementary Materials, Figures S1–S6). The ^13^C NMR spectral data of Ar signals for compounds **1** and **2** and related compounds [32] for comparison purposes are given in Table 1.

The data presented in Table 1 indicate a high level of similarity in the chemical shifts for both series of compounds (i.e., (*p*-FC_6_H_4_)_3_GeX and (*p*-F_3_CC_6_H_4_)_3_GeX), making it difficult to identify them accurately and solely from the NMR spectroscopy data. From this point of view, the investigation of compounds **1**–**4** through XRD analysis may be regarded as an important result for derivatives of the organic group 14 elements.

**Figure 1:**
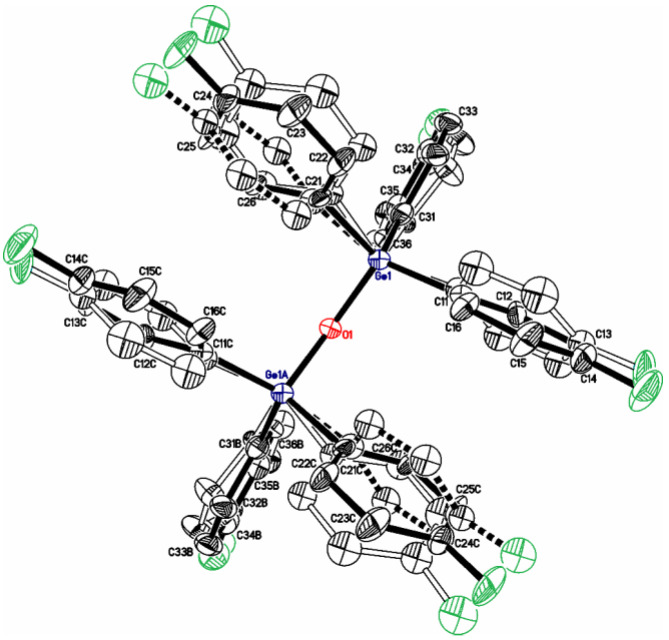
The molecular structure of the [(*p*-FC_6_H_4_)_3_Ge]_2_O (**1**) complex. Displacement ellipsoids are shown at a 30% probability level. Hydrogen atoms have been omitted for clarity. Labels for fluorine atoms and minor components of disorder are not displayed. Minor components of disorder have been drawn using open and dashed lines. The selected bond lengths (Å) and angles (deg) comprised the following: Ge(1)-O(1) 1.7784(17), Ge(1)-C(21A) 1.789(11), Ge(1)-C(11A) 1.838(8), Ge(1)-C(31A) 2.045(11); Ge(1)-O(1)-Ge(1) 132.4(2), O(1)-Ge(1)-C(31A) 99.3(4), O(1)-Ge(1)-C(11A) 110.9(3), O(1)-Ge(1)-C(21A) 114.6(4), C(21A)-Ge(1)-C(11A) 115.4(5), C(21A)-Ge(1)-C(31A) 106.7(6), and C(11A)-Ge(1)-C(31A) 108.4(5).

**Figure 2:**
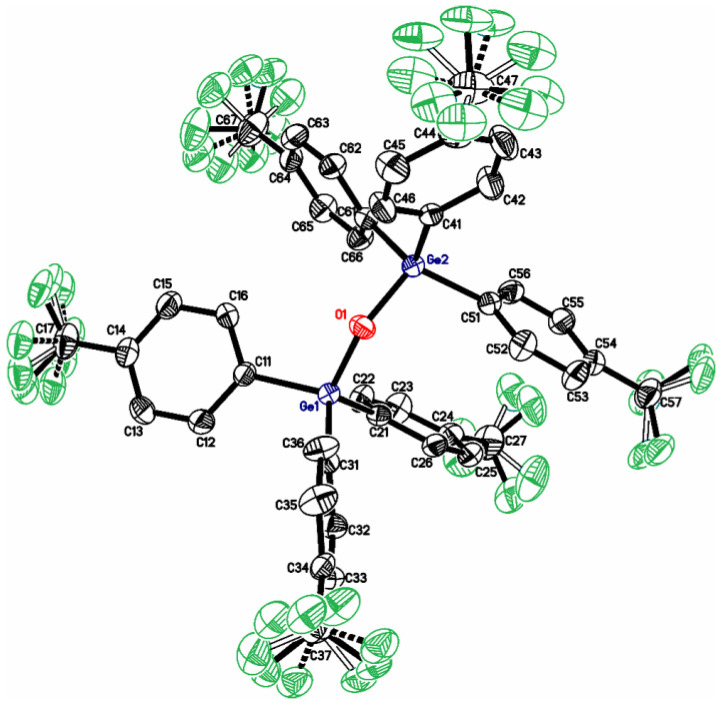
The molecular structure of complex [(*p*-F_3_CC_6_H_4_)_3_Ge]_2_O (**2**). Displacement ellipsoids are shown at a 50% probability level. Hydrogen atoms have been omitted for clarity. Labels for fluorine atoms and minor components of disorder are not displayed. Minor components of disorder have been drawn using open and dashed lines. The selected bond lengths (Å) and angles (deg) comprised the following: Ge(1)-O(1) 1.771(2), Ge(2)-O(1) 1.775(2), Ge(1)-C(31) 1.941(3), Ge(1)-C(21) 1.942(3), Ge(1)-C(11) 1.946(3); Ge(2)-O(1)-Ge(1) 133.63(12), O(1)-Ge(1)-C(31) 104.59(11), O(1)-Ge(1)-C(21) 110.03(11), O(1)-Ge(1)-C(11) 109.32(11), C(21)-Ge(1)-C(11) 110.82(13), C(31)-Ge(1)-C(11) 110.46(13), and C(31)-Ge(1)-C(21) 111.43(12).

**Figure 3:**
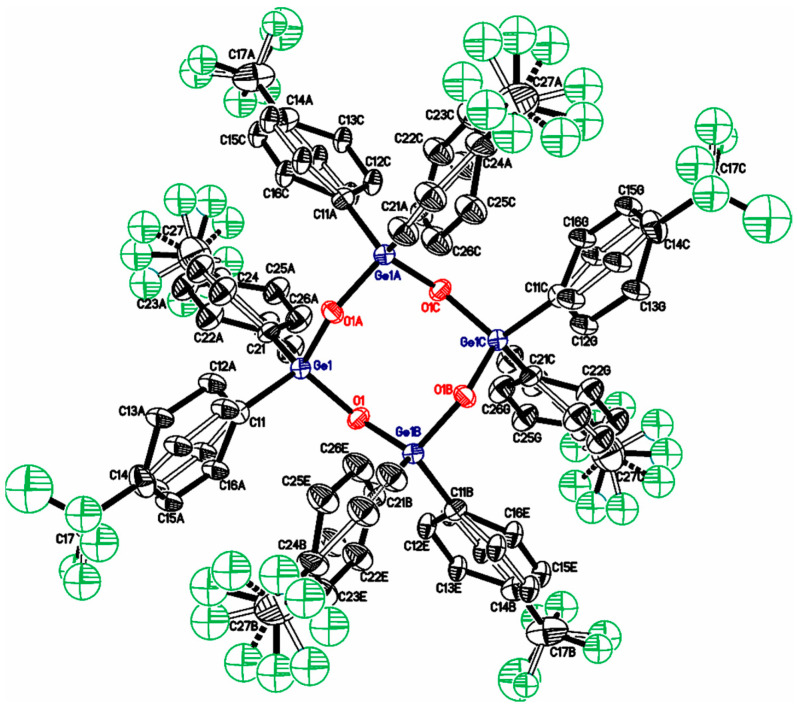
The molecular structure of the *cyclo*-[(*p*-F_3_CC_6_H_4_)_2_GeO]_4_ (**3**) complex. Displacement ellipsoids are shown at a 40% probability level except for the disordered fluorine atoms that were drawn isotropically for clarity. Hydrogen atoms have been omitted for clarity. Labels for fluorine atoms and minor components of disorder are not displayed. Minor components of disorder have been drawn using open and dashed lines. The selected bond lengths (Å) and angles (deg) comprised the following: Ge(1)-O(1) 1.766(6), Ge(1)-O(1A) 1.766(6), Ge(1)-C(11) 1.931(9), and Ge(1)-C(21) 1.934(9); O(1)-Ge(1)-O(1A) 108.8(4), Ge(1)-O(1)-Ge(1A) 124.7(3), O(1A)-Ge(1)-C(11) 107.3(3), O(1)-Ge(1)-C(21) 107.4(3), O(1)-Ge(1)-C(11) 110.6(3), O(1A)-Ge(1)-C(21) 111.0(3), and C(11)-Ge(1)-C(21) 112.7(4).

**Figure 4:**
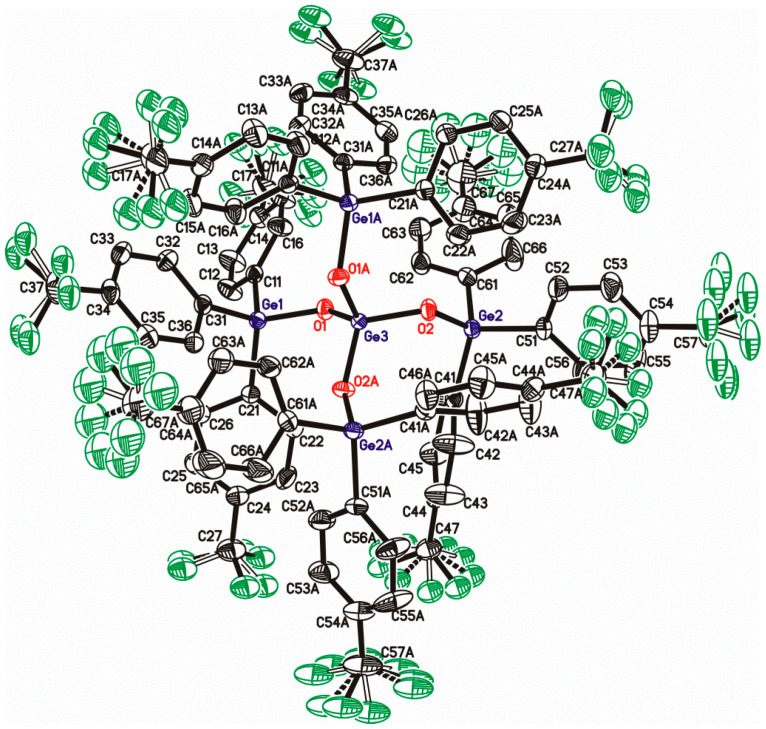
The molecular structure of the [(*p*-F_3_CC_6_H_4_)_3_GeO]_4_Ge (**4**) complex. Displacement ellipsoids are shown at a 40% probability level. Hydrogen atoms and solvated benzene molecules are omitted for clarity. Labels for fluorine atoms and minor components of disorder are not displayed. Minor components of disorder have been drawn using open and dashed lines. The selected bond lengths (Å) and angles (deg) comprised the following: Ge(3)-O(1) 1.730(4), Ge(3)-O(2) 1.735(4), Ge(1)-O(1) 1.781(4), Ge(2)-O(2) 1.781(4), Ge(1)-C(11) 1.936(6), Ge(1)-C(31) 1.948(6), Ge(1)-C(21) 1.951(6), Ge(2)-C(51) 1.936(6), Ge(2)-C(61) 1.938(6), and Ge(2)-C(41) 1.945(6); Ge(3)-O(1)-Ge(1) 132.7(2), Ge(3)-O(2)-Ge(2) 133.8(2), O(1)-Ge(3)-O(1A) 113.7(3), O(1)-Ge(3)-O(2) 107.4(2), O(1)-Ge(3)-O(2A) 107.2(2), O(2)-Ge(3)-O(2A) 114.2(3), O(1)-Ge(1)-C(11) 101.9(2), O(1)-Ge(1)-C(31) 112.2(2), O(1)-Ge(1)-C(21) 109.0(2), C(11)-Ge(1)-C(31) 114.3(3), C(11)-Ge(1)-C(21) 110.1(2), and C(31)-Ge(1)-C(21) 109.1(2).

The compounds studied, **1**–**4**, were isolated as colorless crystals. Their molecular structures, according to the XRD analysis, are shown below (Figure 1, Figure 2, Figure 3 and Figure 4; for details of the XRD experiments that were performed, see Supplementary Materials).

In a crystal of [(*p*-FC_6_H_4_)_3_Ge]_2_O (**1**) (Figure 1), the aryl rings are somewhat disordered due to the differing conformations of the aryl groups. Molecules in the crystal lie on the 2-fold axis passing through the O atom.

**Scheme 3:**
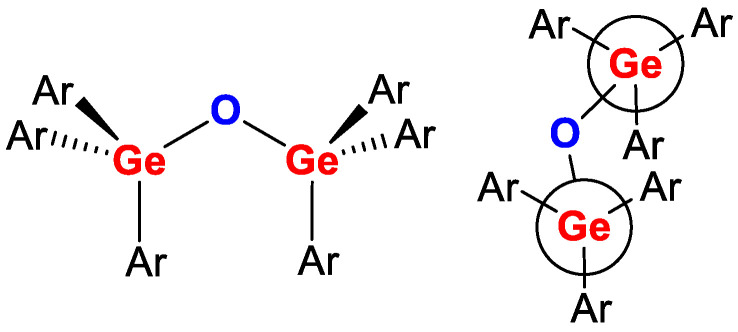
Eclipsed conformation for the germoxanes.

Interestingly, aryl substituents in [(*p*-FC_6_H_4_)_3_Ge]_2_O (**1**) were in an eclipsed conformation (Scheme 3); C_Ar_-Ge-Ge-C_Ar_ torsions were found to be within a 2.4–2.7° range. This was deemed to have been caused by the effects of packing; there was no stacking between the rings on the neighboring Ge atoms. The conformations of two Ar rings on each Ge atom were determined to be propeller-like (C_Ar_-C_Ar_-Ge-O torsions are 26.6/53.0 deg; the ideal torsion is 60 deg), whereas the plane of the third ring was deemed to be near coplanarity with the Ge-O bond (C_Ar_-C_Ar_-Ge-O torsion is 12.5 deg).

In the molecular structure of [(*p*-F_3_CC_6_H_4_)_3_Ge]_2_O (**2**) (Figure 2), all the F_3_C groups were found to be rotationally disordered over two or three positions. It is obvious that such a strong disorder is an immanent property of the crystal packings of this specific class of compounds, and it was therefore decided that they must be displayed in these figures.

The two halves of the molecule in compound **2** were structurally very similar but not equivalent, which could be attributed to crystal packing; as a result, the structural parameters underlying the Ge(1) and Ge(2) atoms slightly differ. The C_Ar_-Ge-Ge-C_Ar_ torsions were in the more typical range of 27.0–38.0°. The torsions at Ge(1) and Ge(2) (30.7/33.0/74.9 and 33.3/51.6/82.5 deg, respectively) were distorted.

In both compounds **1** and **2,** the Ge-O-Ge angle (132.4 and 133.62°, respectively) were more obtuse than in the ideal germoxanes (120°), indicating the presence of an n-σ-conjugation between the O and Ge atoms. Another interesting feature of these compounds is a decrease in the O-Ge-C angle values (99.3 and 104.54°) in comparison with the tetrahedral (ideal value 109.5^o^) ones. Interestingly, in compound **3** (see below), the geometry around the Ge atoms could be described as being more typical.

The molecular structure of *cyclo*-[(*p*-F_3_CC_6_H_4_)_2_GeO]_4_ (**3**) (Figure 3) is characterized by a high disorder of both the F_3_C and aryl groups, resulting in a diminished quality of the XRD experiment. At the same time, the structural parameters were clearly determined.

The eight-membered [GeO]_4_ ring in molecule **3** is in a distorted *boat-chair* [47] conformation, which enables the reduction in the intracyclic tensions between the Ar rings. It should be noted that Bent’s rule was satisfied (angles O(1)-Ge(1)-O(1) 109.0° vs. C(11)-Ge(1)-C(21) 112.5°, respectively), but this small difference may be indicative of the similarity in the electron acceptor properties of the O and *p*-F_3_CC_6_H_4_ groups on the Ge atoms. 

Unlike structures **1**–**3**, three types of the non-equivalent Ge atoms, the central GeO_4_, and the terminal GeC_3_O (with a 2-fold molecular symmetry) were observed in [(*p*-F_3_CC_6_H_4_)_3_GeO]_4_Ge (**4**) (Figure 4). This leads, for instance, to the difference in the Ge-O bond lengths; thus, for the central GeO_4_ fragment, this distance is smaller than for the organogermanium C_3_Ge-O node (1.733 vs. 1.781 Å, respectively), which, in classical cases, may be explained by the introduction of a more sterically huge ligand. Similar to compounds **1** and **2**, the Ge-O-Ge angles in compound **4** were found to be obtuse (132.73 and 133.86°), which may indicate a high degree of conjugation between the Ar groups and the Ge and O atoms, which provides a perspective for the design and synthesis of functionalized materials.

In general, the molecule of compound **4** loses its high symmetry; small differences in the structural parameters were observed, which could be attributed to the packing forces. The conformations of the Ar rings along the Ge-O bond were found to be different for the two types of Ge atoms: Ge(2) may be characterized as a real propeller-like atom (C_Ar_-C_Ar_-Ge-O torsions are 39.2/55.2/56.5 deg), whereas Ge(1) is distorted (C_Ar_-C_Ar_-Ge-O torsions are 10.0/35.6/75.8 deg).

It is interesting to note that compounds **1** and **2** crystallize in the monoclinic crystal system, with the *C*2/c and *P*2_1_/c space groups, respectively. Compounds **3** and **4,** in crystals, are characterized by the tetragonal system (with the *I*4_1_/a and *P*4_3_2_1_2 space groups, respectively). As a unique property of the compounds studied, we should add that the achiral compound [(*p*-F_3_CC_6_H_4_)_3_GeO]_4_Ge (**4**) was crystallized in a chiral crystal (with the *P*4_3_2_1_2 Sohncke space group, Z = 4); the Flack parameter was 0.001(13). The emerging chirality of the crystal was attributed to the spiralization of the Ar rings (their propeller-like “angulation” in one direction), while the presence of the central “symmetric” GeO_4_ core also seemed to be important. This allows a cautious conclusion to be made that the modification of the substituents in the aryl groups (especially, in *ortho*-positions to the Ge atom) may, in future works, lead to a more pronounced chirality for the germoxanes, which is promising for the design of chiral materials.

As a common feature for compounds **1**–**4**, the geometry of the germanium atoms in a crystal may be described as a distorted tetrahedral. As an additional structural feature of the germoxanes, we could describe the Ge…Ge separation in the structures of compounds **1**–**4**. It is evident that this distance strongly depends on the Ge-O-Ge angle, where greater angle values result in an increase of Ge…Ge separation, which was evident when comparing the compounds **1** (3.255 Å; 132.4°), **2** (3.261 Å; 133.6°), **3** (3.130 Å; 124.5°) and **4** (3.217/3.235 Å; 132.4/133.86°). Other factors may also had an impact, as was evident in the case of compound **4**; first and foremost, the steric size of their substituents on the Ge atoms; their decrease at the central Ge atom resulted in a decrease in the Ge…Ge separation. The packing effects in a crystal are also taken into account.

## 3. Discussion

**Scheme 4:**
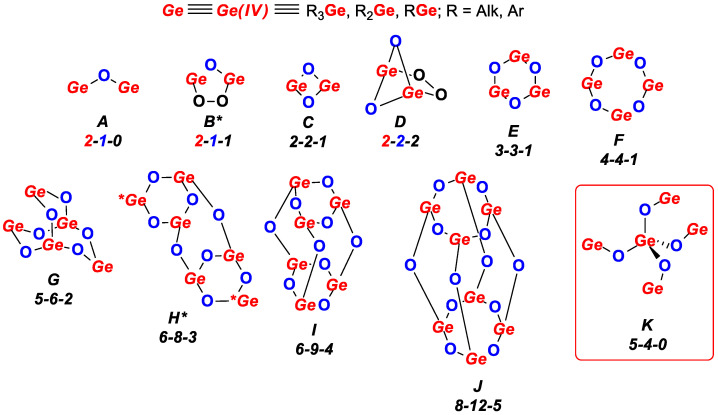
Organogermanium Ge-O derivatives of the known structural types (**A**–**J**) and those that were first established in this work (**K**). These structures are classified by three numbers indicating the number of Ge atoms, that of the O atoms, and the number of cycles.

Analysis of the known structures of organogermanium compounds with Ge-O bonds enabled the formulation of 10 structural crystal types, **A**–**J** (Scheme 4). They differ in three main features: (1) the number of Ge atoms; (2) that of the O atoms; and (3) the number of intramolecular cycles. These known structures are also presented in Table 2. It is necessary to say that we added two structural types to the carbon-substituted germanes, containing other groups: (1) B* represents the OH-substituted derivatives; and (2) H* represents the partially heteroatom-substituted compounds (i.e., *Ge atoms contain Cl or OH) with a ladder-type structure. We added these unusual structural types to the full representation of a wide variety of Ge-O species. In general, it could be stated that compounds of the H*, **I**, and **J** structures are referred to as the cage type, and **I** and **J** are germasesquioxides. Analysis of these germoxane structures through XRD analysis is especially important, as the Ge nucleus (^73^Ge) is not a good object for solution NMR spectroscopy due to its low content and quadrupole moment.

The molecular structures of the germoxanes **1**, **2**, and **3** refer to the known types **A** and **F**, respectively. Moreover, the structural parameters of **1**–**3** are close to the known ones (see Table 2 below). In contrast, the molecular structure of **4** represents a novel type of Ge-O species (type **K**, Scheme 4).

Analyzing the types of the germoxanes that were formed under the hydrolysis of R_2_GeHal_2_, it is important to note that the formation of four-membered cycles, **C**, is possible in the case of the voluminous substituents at the Ge atoms; otherwise, the tendency of the six- and eight-membered cycles, **E** and **F**, respectively, was observed. The predominance of the six- or eight-membered cycles depends on the steric size of the R substituents, whereas a smaller R favorably gives larger rings [48] with significant amounts of polymers. Draeger et al. [46] stated that the phenyl-substituted derivatives *cyclo*-[Ph_2_GeO]_4_ and [Ph_2_GeO]_n_ could be thermally (228–263 °C) transferred into more thermodynamically stable *cyclo*-[Ph_2_GeO]_3_. Thus, the germoxanes of large cycles were determined to be kinetic products that were formed under mild conditions in diluted solutions, which was observed, in this case, for *cyclo*-[(*p*-F_3_CC_6_H_4_)_2_GeO]_4_ (**3**).

**Table 2:** The main structural types of the organogermanium Ge-O derivatives (according to XRD analysis; structural features are given for the Scheme 4 bonds).

Structural Type	Linear Formula of the Ge-Containing Fragment	CCDS Code	Main Structural Parameters (Average Values)			Reference
			*d*(Ge-O), Å	Ge-O-Ge Angle, deg	O-Ge-O Angle, deg	
**A**	1,1′-Fc[*cyclo*-GeC_4_Me_4_]_2_O	BIPVOL	1.752	136.5	-	[49]
	[(PhCH_2_)_3_Ge]_2_O	BZGEOX10	1.730	180.0	-	[50]
	[Mes_2_(2-Flu)Ge]_2_O	KIXWAP	1.787	151.0	-	[51]
	[1,1′-Naphth-2-GeMe_2_]_2_O	ROGMAB	1.789	119.6	-	[52]
	[Ph_3_Ge]_2_O	TPGERO	1.752	137.9	-	[53]
	[Ph_3_Ge]_2_O	TPGERO01	1.767	135.2	-	[54]
	[(F_5_C_2_)_3_Ge]_2_O	UKUBAF	1.742	150.2	-	[31]
	*[(p-FC_6_H_4_)_3_Ge]_2_O* (**1**)	*-*	*1.778*	*132.4*	*-*	*This work*
	*[(p-F_3_CC_6_H_4_)_3_Ge]_2_O* (**2**)	*-*	*1.774*	*133.6*	*-*	*This work*
**B**	*trans-cyclo*-[DippGe(OH)O]_2_O	CEDNIJ	1.752	110.8	-	[55]
**C**	*cyclo*-[*cyclo*-((CH_2_C(SiMe_3_)_2_)_2_Ge)O]_2_	EYEBEP	1.821	94.5	85.5	[56]
	*cyclo*-[Mes_2_GeO]_2_	MEYPEL	1.812	92.0	88.0	[40]
	*cyclo*-[(2,6-Et_2_C_6_H_3_)_2_GeO]_2_	VALKIC	1.817	92.1	87.6	[39]
	*trans-cyclo*-[Fc(Tip)GeO]_2_	WEPDAY	1.816	92.8	87.2	[57]
	*trans*-*cyclo*-[Mes(Tbt)GeO]_2_*C_6_H_14_	XIXNUN	1.813	94.2	85.7	[41]
	*trans*-*cyclo*-[Mes(Tbt)GeO]_2_	XIXPAV	1.810	96.0	84.0	[41]
	*cis*-*cyclo*-[Mes(Tbt)GeO]_2_	XIXPEZ	1.810	92.2	86.1	[41]
**D**	*bicyclo*-[2,6-Dipp_2_C_6_H_3_Ge(O)O]_2_	XAMQIN	1.802	84.2	87.1	[42]
**E**	*cyclo*-[Ph_2_GeO]_3_	BECHEW	1.769	128.6	107.4	[33]
	*cyclo*-[(*t*Bu)_2_GeO]_3_	BUDVIF	1.781	133.2	106.8	[58]
	*cyclo*-[(*t-*Bu)_2_GeO]_3_	BUDVIF10	1.781	133.0	107.0	[34]
	*cyclo*-[(Me_4_Si(O)(CH_2_)_2_Ge)O]_3_	DUBZEF	1.758	127.2	109.4	[59]
	*cyclo*-[Ph_4_C_4_GeO]_3_	EGULAT	1.768	132.5	107.6	[35]
	*cyclo*-[(F_5_C_2_)_2_GeO]_3_*phen ^a^	EKEGOS	1.735	125.7	113.5	[37]
	*cis-cyclo*-[Fc(Ph)GeO]_3_	VAHNUO	1.771	127.5	107.8	[36]
	*trans-cyclo*-[Fc(Ph)GeO]_3_	VAHPAW	1.776	123.2	105.9	[36]
**F**	*cyclo*-[Ph_2_GeO]_4_	CIRBIO	1.756	134.2	109.0	[46]
	*cyclo*-[(2,8-Et_2_C_8_H_2_Ge)O]_4_	PAFHEM	1.762	126.96	105.9	[23]
	*cyclo*-[(CH_2_)_5_GeO]_4_	SONHAE	1.775	125.8	107.7	[60]
	*cyclo-[(p-F_3_CC_6_H_4_)_2_GeO]_4_* (**3**)	*-*	*1.769*	*124.5*	*109.0*	*This work*
**G**	*bicyclo*-[PhGe(Ph_2_GeO_2_)_3_GePh]	CUPHIE	1.76	130.9	109.1	[43]
H*	*trans-tricyclo*-[(*t*-BuGe)_6_O_8_Cl_2_]	FOTCAS	1.76	129.7	107.7	[61]
	*trans-tricyclo*-[(*t*-BuGe)_6_O_8_Cl_2_]	FOTCAS01	1.76	126.5	107.0	[62]
	*tricyclo*-[(Me_2_NCH_2_FcGe)_6_O_8_(OH)_2_]	MUSYUU	1.766	125.6	108.2	[45]
**I**	*tetracyclo*-[(*t*-BuGe)_6_O_9_]	CATFEI	1.750	132.7	108.3	[63]
	*tetracyclo*-[(MesGe)_6_O_9_]	GIHSAR	1.751	131.0	107.8	[64]
	*tetracyclo*-[(*i*-PrGe)_6_O_9_]	GIHSEV	1.760	129.7	108.4	[64]
	*tetracyclo*-[(*i*-PrGe)_6_O_9_]	GIHSEV01	1.755	130.5	108.0	[65]
	*tetracyclo*-[(*i*-PrGe)_6_O_9_]	GIHSEV02	1.756	131.0	108.0	[62]
	*tetracyclo*-[(CyGe)_6_O_9_]	GIHSIZ	1.748	131.9	108.5	[64]
	*tetracyclo*-[(CyGe)_6_O_9_]	GIHSIZ01	1.758	133.3	108.6	[62]
**J**	*pentacyclo*-[(VinSiMe_2_OGeO)_8_O_4_]*Et_4_NF	VOFZIC	1.721	133.8	113.9	[12]
***K***	*[(p-F_3_CC_6_H_4_)_3_GeO]_4_Ge* (**4**)	*-*	*1.757*	*133.3*	*110.6*	*This work*

^a^ Tetracoordinated Ge atom is described.

As evident from Table 2, the Ge-O bond length in germoxanes varies within the range of 1.73–1.82 Å irrespective of whether a cyclic or acyclic species is formed, whereas a slight increase was observed for the four-membered cycles, **C,** due to the ring-bond angles’ strains. Introduction of sterically voluminous substituents to the Ge atom also resulted in some lengthening of the Ge-O bonds. The average value for *d*(Ge-O) was 1.761 Å. Related tendencies were observed for the bond angles; thus, the angle values were more sensitive to cycle formation, and, as is evident, for the small-membered cyclic structures (like **C** and **D**), they were determined to be smaller compared to the acyclic species (Ge-O-Ge 84–96° vs. 119–180°, respectively). The germoxane [(PhCH_2_)_3_Ge]_2_O, with a Ge-O-Ge angle of 180 deg, is a typical example of the effect of the steric volumes underlying the substituents. As a rule, the general value of the Ge-O-Ge angle in acyclic compounds is 130.6°. The O-Ge-O angles are sensitive to the steric sizes of the substituents on the Ge atom; an increase in their sizes resulted in an increase of these geometric parameters. Its typical value is 108.4 deg.

Introduction of the aryl electron-withdrawing groups in compounds **1** and **2** in comparison with [Ph_3_Ge]_2_O resulted in a weak Ge-O bond elongation (1.774–1.778 vs. 1.752–1.767 Å, respectively); the Ge-O-Ge angles were expectedly decreased (132.4–133.6 vs. 135.2–137.9°, respectively), indicating a σ-n-conjugation between the Ge and O atoms. The same features were observed when comparing *cyclo-*[(*p*-F_3_CC_6_H_4_)_2_GeO]_4_ (**3**) with *cyclo*-[Ph_2_GeO]_4_.

Compound **4** may be regarded as a molecular analog of inorganic germanates, the precursor of germanium zeolites. The presence of organic substituents at peripheral Ge atoms in compound **4** enables the formation of chiral monocrystals that are suitable to undergo XRD analysis. It is known that germanium zeolites [66] and molecular Ge compounds [67] of similar structures may be used for the sorption of hydrocarbons. Special interest will be paid to the organogermanium compounds, including these and other (absorption/emission optical [68] and semiconducting [69]) properties caused by the different types of conjugation (σ-, π-, and d-) [70] between the structural blocks, which stimulates in future novel research on germanium-heteroatom derivatives.

## 4. Materials and Methods

***General methods and instrumentation.*** ^1^H NMR (400.130 MHz), ^13^C NMR (100.613 MHz), and ^19^F (376.498 MHz) spectra were recorded with either a Bruker 400 or Agilent 400 spectrometer at 295 K. Chemical shifts were given in ppm relative to internal Me_4_Si (^1^H and ^13^C NMR spectra) or external CFCl_3_ (^19^F spectra). Elemental analyses were carried out using a HeraeusVarioElementar instrument. MALDI mass spectra were recorded on an Autoflex II Bruker spectrometer (fwhm resolution: 18,000) equipped with a nitrogen laser operating at a wavelength of 337 nm and with a time-of-flight mass analyzer operating in reflection mode. The accelerating voltage was 20 kV, and anthracene (“Acros”, 99%) was used as the matrix.

***Starting materials.*** The initial compounds (*p*-FC_6_H_4_)_3_GeBr and (*p*-F_3_CC_6_H_4_)_3_GeBr were obtained following the procedure outlined in the literature [32]. Other compounds that were used, including GeCl_4_ and NaOH (“Aldrich”), are commercially available and were used as received. Deionized water was used for hydrolysis. Solvents were purified using the usual procedures. *n*-Hexane was refluxed over sodium and then distilled off. THF was refluxed and distilled off over KOH. Deuterated solvent (CDCl_3_) was dried over CaH_2_, distilled, and stored under argon. 

***General procedure for the synthesis of the target compounds [(p-FC_6_H_4_)_3_Ge]_2_O *(1)* and [(p-F_3_CC_6_H_4_)_3_Ge]_2_O *(2)*.*** NaOH powder (0.41 mmol, 2 eq.) was added to the solution of Ar_3_GeBr (0.20 mmol) in THF (8 mL) at 0 °C; then, H_2_O (0.20 mL, 11.10 mmol, and 55.6 eq.) was added. The reaction mixture was stirred at room temperature for 48 h. Following this, all the volatile materials were removed under a reduced pressure; the residue was dissolved in ether (20 mL), washed with water (3 × 10 mL), and dried over Na_2_SO_4_. After evaporation, the residue was recrystallized from the minimal amount of an *n*-heptane/CH_2_Cl_2_ mixture to yield the crystal materials.

***Germoxane [(p-FC_6_H_4_)_3_Ge]_2_O *(1)*.*** Yield: 0.0615 g (84%). Colorless prisms. 

^1^H NMR (δ, ppm, CDCl_3_): 7.58–7.52, 7.16–7.10 (2 m, each 12H, Ar_H_). 

^13^C NMR (δ, ppm, CDCl_3_): 164.37 (d, *^1^J*_13C-19F_ 250.3 Hz, *ipso*-Ar_C_), 135.93 (d, *^3^J*_13C-19F_ 7.6 Hz, *o*-Ar_CH_), 130.81 (d, *^4^J*_13C-19F_ 3.8 Hz, *ipso*-Ar_CGe_), and 115.92 (d, *^2^J*_13C-19F_ 19.8 Hz, *m*-Ar_CH_).

^19^F NMR (δ, ppm, CDCl_3_): −109.53–(−109.60) (m, 6F, 6 *p*-C_6_H_4_F). 

MALDI, *m*/*z* (rel %): 732 ([M]^+^, 100). 

Anal. calcd for C_36_H_24_F_6_Ge_2_O (731.8456): C, 59.08; H, 3.31. Found: C, 58.86; H, 3.22.

***Germoxane [(p-F_3_CC_6_H_4_)_3_Ge]_2_O *(2)*.*** Yield: 0.0887 g (86%). Colorless blocks.

^1^H NMR (δ, ppm, CDCl_3_): 7.55 and 7.50 (2d, ^3^*J*_H-H_ 8.1 Hz, each 12H, Ar_H_). 

^13^C NMR (δ, ppm, CDCl_3_): 139.76 (*ipso*-Ar_CGe_), 134.21 (*o*-Ar_CH_), 132.59 (q, ^2^*J*_13C-19F_ 32.6 Hz, *ipso*-Ar_C_), 125.25 (q, ^3^*J*_13C-19F_ 3.7 Hz, *m*-Ar_CH_), and 123.69 (q, ^1^*J*_13C-19F_ 271.3 Hz) (CF_3_). 

^19^F NMR (δ, ppm, CDCl_3_): −63.34 (s, 18F, 6 *p*-C_6_H_4_CF_3_). 

MALDI, *m*/*z* (rel %): 1032 ([M]^+^, 100). 

Anal. calcd for C_42_H_24_F_18_Ge_2_O (1031.8906): C, 48.89; H, 2.34. Found: C, 48.78; H, 2.41.

***Target compounds cyclo-[(p-F_3_CC_6_H_4_)_2_GeO]_4_ *(3)*, [(p-F_3_CC_6_H_4_)_3_GeO]_4_Ge *(4)*.*** Compounds **3** and **4** were obtained in trace amounts under the slow crystallization of the mother liquids (*n*-hexane or *n*-hexane/benzene mixture) during the synthesis of the germanes (*p*-FC_6_H_4_)_3_GeHal and (*p*-F_3_CC_6_H_4_)_3_GeHal (Hal = Cl or Br), as described by Zaitsev et al. [32], based on the standard procedure (98.00 mmol of GeCl_4_).

***Germoxane cyclo-[(p-F_3_CC_6_H_4_)_2_GeO]_4_ *(3)*.*** Yield: 0.0179 g (<1% based on GeCl_4_), trace amounts). Colorless plates. 

MALDI, *m*/*z* (rel %): 1515 ([M]^+^, 100). 

Anal. calcd for C_56_H_32_F_24_Ge_4_O_4_ (1515.3726): C, 44.39; H, 2.13. Found: C, 44.54; H, 2.22.

***Germoxane [(p-F_3_CC_6_H_4_)_3_GeO]_4_Ge *(4)*.*** Yield: 0.0043 g (<1% based on GeCl_4_, trace amounts). Colorless blocks. 

MALDI, *m*/*z* (rel %): 2168 ([M]^+^, 100). 

Anal. calcd for C_84_H_48_F_36_Ge_5_O_4_*2C_6_H_6_ (2324.6437): C, 49.60; H, 2.60. Found: C, 49.24; H, 2.48.

***X-ray diffraction study.*** Experimental intensities were collected on a Bruker Smart Apex II diffractometer (for compounds **1**, **3**, and **4**) and a Gemini Ultra machine (for compound **2**) using graphite monochromatized Mo-*K*α radiation (λ = 0.71073 Å). Absorption corrections based on measurements of the equivalent reflections were applied. The structures were solved using direct methods and refined with the full matrix least-squares on *F*^2^ (Shelxtl) with anisotropic thermal parameters for all non-hydrogen atoms except for the disordered -CF_3_ groups; they were refined with restrained C–F and F…F distances. In compounds **1** and **3**, benzene rings were found to be disordered over two or three positions; they were refined using FLAT instructions. All H atoms were placed in calculated positions and refined using a riding model. Details are listed in Tables S1–S3, Supporting Information. Crystallographic data have been deposited with the Cambridge Crystallographic Data Centre as supplementary publication no. 2281822–2281825.

## 5. Conclusions

Germanium compounds with Ge-O-Ge bonds may be regarded as organic models of inorganic GeO_2_ or anionic [OGe(O)_2_]_n_^2n−^ species, making these compounds convenient for investigation. The structural features of Ge-O derivatives described in this work are important for the design and construction of novel materials, including porous, nanostructured (cages, ladders etc.), optoelectronic, and chiral compounds. Substitution of the silicon compounds with the germanium analogs may be promising in the design of new materials with improved physical properties. An important result of the work was the establishment of the structural features of four germoxanes with aryl electron-withdrawing substituents, indicating the impact of the Ar group on the structural characteristics. The characterization of germoxane **K** on the novel structural type has prospects in the construction of novel Ge-O-Ge compounds.

## Figures and Tables

**Table 1:** Aromatic (Ar_C_) ^13^C NMR spectral signals for [(*p*-FC_6_H_4_)_3_Ge]_2_O (**1**), [(*p*-F_3_CC_6_H_4_)_3_Ge]_2_O (**2**), and related (*p*-FC_6_H_4_)_3_GeX, and (*p*-F_3_CC_6_H_4_)_3_GeX compounds (X = Cl, Br, or H) (in ppm, CDCl_3_) ^a^.

Compound	X	*ipso*-Ar_C_	*o*-Ar_CH_	*ipso*-Ar_CGe_	*m*-Ar_CH_
(*p*-FC_6_H_4_)_3_GeX	X = Cl	164.59 (251.0)	136.00 (8.1)	129.73 (3.7)	116.15 (20.8)
	X = Br	164.52 (251.0)	136.11 (8.1)	129.64 (3.3)	116.09 (20.5)
	X = H	163.87 (248.7)	136.75 (7.6)	130.22 (3.8)	115.75 (20.6)
*[(p-FC_6_H_4_)_3_Ge]_2_O* (**1**) *(this work)*		*164.37 (250.3)*	*135.93 (7.6)*	*130.81 (3.8)*	*115.92 (19.8)*
(*p*-F_3_CC_6_H_4_)_3_GeX	X = Cl	133.86 (32.8)	133.13	138.74	125.76 (3.8)
	X = Br	133.11 (32.9)	134.49	137.80	125.60 (3.7)
	X = H	131.98 (32.5)	135.36	138.65	125.25 (3.7)
*[(p-F_3_CC_6_H_4_)_3_Ge]_2_O* (**2**) *(this work)*		*132.59 (32.6)*	*134.21*	*139.76*	*125.25 (3.7)*

^a^ Data for the known compounds were obtained from the data published by the authors of [32]; values in parentheses indicate the spin coupling constant *J*_13C-19F_ (in Hz).

## Data Availability

Not applicable.

## Supplementary Materials

The following supporting information can be downloaded at: https://www.mdpi.com/article/10.3390/ijms241713575/s1.

## References

[1] Mazurek P., Vudayagiri S., Skov A.L. (2019). How to tailor flexible silicone elastomers with mechanical integrity: A tutorial review. Chem. Soc. Rev..

[2] Köhler T., Gutacker A., Mejía E. (2020). Industrial synthesis of reactive silicones: Reaction mechanisms and processes. Org. Chem. Front..

[3] Cordes D.B., Lickiss P.D., Rataboul F. (2010). Recent Developments in the Chemistry of Cubic Polyhedral Oligosilsesquioxanes. Chem. Rev..

[4] Unno M., Suto A., Matsumoto T. (2013). Laddersiloxanes—Silsesquioxanes with defined ladder structure. Russ. Chem. Rev..

[5] Kamino B.A., Bender T.P. (2013). The use of siloxanes, silsesquioxanes, and silicones in organic semiconducting materials. Chem. Soc. Rev..

[6] John Ł., Ejfler J. (2023). A Brief Review on Selected Applications of Hybrid Materials Based on Functionalized Cage-like Silsesquioxanes. Polymers.

[7] Skoczeń A., Frąckowiak D., Przekop R.E., Frydrych M., Kasperkowiak M., Jeleń P., Sitarz M., Marciniec B. (2022). New Ceramics Precursors Containing Si and Ge Atoms—Cubic Germasilsesquioxanes—Synthesis, Thermal Decomposition and Spectroscopic Analysis. Molecules.

[8] Smart M.M., McMillen C.D., Ivey K., Kolis J.W. (2020). Chemistry of Metal Silicates and Germanates: The Largest Metal Polygermanate, K_11_Mn_21_Ge_32_O_86_(OH)_9_(H_2_O), with a 76 Å Periodic Lattice. Inorg. Chem..

[9] Lin Z.-E., Yang G.-Y. (2010). Germanate Frameworks Constructed from Oxo Germanium Cluster Building Units. Eur. J. Inorg. Chem..

[10] Huang S., Yue H., Chen Y., Liu Y., Guan Y., Zou X., Sun J. (2018). Three-Dimensional Open-Framework Germanate Built from a Novel Ge13 Cluster and Containing Two Types of Chiral Layers. Cryst. Growth Des..

[11] Villaescusa L.A., Lightfoot P., Morris R.E. (2002). Synthesis and structure of fluoride-containing GeO_2_ analogues of zeolite double four-ring building units. Chem. Commun..

[12] Sato N., Hayashi T., Tochigi K., Wada H., Shimojima A., Kuroda K. (2019). Synthesis of Organosilyl-Functionalized Cage-Type Germanoxanes Containing Fluoride Ions. Chem. Eur. J..

[13] Stock N., Jargstorff C., Wriedt S. (2011). Two New Crystalline Organogermanate-Based Inorganic-Organic Hybrid Compounds. Z. Anorg. Allg. Chem..

[14] Hayashi T., Murase N., Sato N., Fujino K., Sugimura N., Wada H., Kuroda K., Shimojima A. (2022). Fluoride Ion-Encapsulated Germoxane Cages Modified with Organosiloxane Chains as Anionic Components of Ionic Liquids. Organometallics.

[15] He H., Cao G.-J., Zheng S.-T., Yang G.-Y. (2009). Lanthanide Germanate Cluster Organic Frameworks Constructed from {Ln_8_Ge_12_} or {Ln_11_Ge_12_} Cage Cluster Building Blocks. J. Am. Chem. Soc..

[16] Hayashi T., Sato N., Wada H., Shimojima A., Kuroda K. (2021). Variation of counter quaternary ammonium cations of anionic cage germanoxanes as building blocks of nanoporous materials. Dalton Trans..

[17] Endo M., Fugami K., Enokido T., Sano H., Kosugi M. (2007). Palladium-Catalyzed Cross-Coupling Reaction Using Arylgermanium Sesquioxide. Adv. Synth. Catal..

[18] Pitteloud J.-P., Zhang Z.-T., Liang Y., Cabrera L., Wnuk S.F. (2010). Fluoride-Promoted Cross-Coupling of Chloro(mono-, di-, or triphenyl)germanes with Aryl Halides in “Moist” Toluene. Multiple Transfer of the Phenyl Group from Organogermane Substrates and Comparison of the Coupling Efficiencies of Chloro(phenyl)germanes with their Corresponding Stannane and Silane Counterparts. J. Org. Chem..

[19] Nakamura T., Shimada Y., Sato K., Lee V.Y. (2023). Bioorganic and Medicinal Organogermanium Chemistry. Organogermanium Compounds: Theory, Experiment, and Applications.

[20] Duverneuil G., Mazerolles P., Perrier E. (1995). Polygermoxanes suitable for biochemical purposes: II Linear trigermoxanes (high-viscosity oils). Appl. Organomet. Chem..

[21] Levitsky M.M., Bilyachenko A.N., Shubina E.S. (2019). Cagelike metallagermanates and metallagermoxanes: Synthesis, structures and functional properties. Coord. Chem. Rev..

[22] Liu J., Ren X., Yan Y., Wang N., Wang S., Zhang H., Li J., Yu J. (2018). A new two-dimensional layered germanate with in situ embedded carbon dots for optical temperature sensing. Inorg. Chem. Front..

[23] Ohshita J., Nakamura M., Ooyama Y. (2015). Preparation and Reactions of Dichlorodithienogermoles. Organometallics.

[24] Zaitsev K.V., Kapranov A.A., Churakov A.V., Poleshchuk O.K., Oprunenko Y.F., Tarasevich B.N., Zaitseva G.S., Karlov S.S. (2013). “Donor–Acceptor” Oligogermanes: Synthesis, Structure, and Electronic Properties. Organometallics.

[25] Hemmert C., Gornitzka H., Lee V.Y. (2023). X-ray Crystallography of Organogermanium Compounds. Organogermanium Compounds: Theory, Experiment, and Applications.

[26] Zaitsev K.V., Oprunenko Y.F. (2021). Reaction of Substituted Group 14 Element Potassium Salts with 1-(Chloromethyl)silatrane: Substitution or Rearrangement?. Russ. J. Gen. Chem..

[27] Zaitsev K.V., Poleshchuk O.K., Churakov A.V. (2022). Oligoorganogermanes: Interplay between Aryl and Trimethylsilyl Substituents. Molecules.

[28] Zaitsev K.V., Trubachev A.D., Poleshchuk O.K. (2023). Germanium Complexes with ONO Tridentate Ligands: O-H Bond Activation Control According to DFT Calculations. Int. J. Mol. Sci..

[29] Zaitsev K.V., Lee V.Y. (2023). Organogermanium Compounds of the Main Group Elements. Organogermanium Compounds: Theory, Experiment, and Applications.

[30] Delouche T., Roisnel T., Dorcet V., Hissler M., Bouit P.-A. (2021). Mixing Polyaromatic Scaffolds and Main Group Elements: Synthesis, Coordination and Optical Properties of Naphthyl-Fused Heteropines. Eur. J. Inorg. Chem..

[31] Pelzer S., Neumann B., Stammler H.-G., Ignat’ev N., Hoge B. (2016). Synthesis of Tris(pentafluoroethyl)germanes. Chem. Eur. J..

[32] Zaitsev K.V., Lam K., Zhanabil Z., Suleimen Y., Kharcheva A.V., Tafeenko V.A., Oprunenko Y.F., Poleshchuk O.K., Lermontova E.K., Churakov A.V. (2017). Oligogermanes Containing Only Electron-Withdrawing Substituents: Synthesis and Properties. Organometallics.

[33] Roß L., Dräger M. (1982). Über Germanium-haltige Heterocyclen, IV. 2,2,4,4,6,6-Hexaphenyl-1,3,5-trioxa-2,4,6-trigermacyclohexan, (Ph_2_GeO)_3_, eine monoplanare Zwischenstufe im Pseudorotationskreislauf der Twist-Wanne-6-Ring-Konformation. Chem. Ber..

[34] Puff H., Franken S., Schuh W., Schwab W. (1983). Darstellung und struktur von di-t-butylgermaniumdi-hydroxid und di-t-butylgermaniumoxid. J. Organomet. Chem..

[35] Godelie M.C., Jennings M.C., Baines K.M. (2001). The Synthesis and Characterization of the Cyclotrigermoxane: (Ph_4_C_4_GeO)_3_. Main Group Met. Chem..

[36] Zhang Y., Cervantes-Lee F., Pannell K.H. (2003). Hydrolysis of tert-Butyl and Phenyl Ferrocenyldichlorogermanes:  Characterization of Di-tert-butyldiferrocenyldigermoxanediol and 1,3,5-Triphenyl-1,3,5-triferrocenylcyclotrigermoxane. Organometallics.

[37] Pelzer S., Neumann B., Stammler H.-G., Ignat’ev N., Hoge B. (2016). Synthesis of Bis(pentafluoroethyl)germanes. Chem. Eur. J..

[38] Glockling F., Houston R.E. (1973). Reactions of organodigermanes. J. Chem. Soc. Dalton Trans..

[39] Masamune S., Batcheller S.A., Park J., Davis W.M., Yamashita O., Ohta Y., Kabe Y. (1989). Oxygenation of digermene derivatives. J. Am. Chem. Soc..

[40] Samuel M.S., Jennings M.C., Baines K.M. (2001). The addition of oxygen to tetramesityldigermene. J. Organomet. Chem..

[41] Tokitoh N., Kishikawa K., Okazaki R., Sasamori T., Nakata N., Takeda N. (2002). Synthesis and characterization of an extremely hindered tetraaryl-substituted digermene and its unique properties in the solid state and in solution. Polyhedron.

[42] Wang X., Peng Y., Olmstead M.M., Fettinger J.C., Power P.P. (2009). An Unsymmetric Oxo/Imido-Bridged Germanium-Centered Singlet Diradicaloid. J. Am. Chem. Soc..

[43] Häberle K., Dräger M. (1984). Germanium-haltige Heterocyclen, VI [1] Ph_8_Ge_5_O_6_, ein Germoxan aus bicyclisch verknüpften (GeO)_4_-Ringen. Z. Naturforsch. B.

[44] Beckmann J., Jurkschat K., Schürmann M. (2000). On the Hydrolysis of *t*Bu_2_Ge(OEt)_2_: Supramolecular Self Assembly in the Solid State of 2 *t*Bu_2_Ge(OH)_2_, (*t*Bu_2_GeOH)_2_O, and H_2_O. Eur. J. Inorg. Chem..

[45] Lorenz V., Jacob K., Wagner C., Görls H. (2002). Metalloxane des Siliciums und Germaniums mit dem 2-(Dimethylaminomethyl)ferrocenyl-Liganden (FcN): Darstellung und Molekülstrukturen von (FcN)_4_M_4_O_4_(OH)4(M = Si, Ge), (FcN)_6_Ge_6_O_8_(OH)_2_ und von (FcN)_2_Si(OH)_2_. Z. Anorg. Allg. Chem..

[46] Roß L., Dräger M. (1984). Formen des Diphenylgermaniumoxids(Ph_2_GeO)_x_, x = 3, 4, n/Phases of Diphenylgermanium Oxide(Ph_2_GeO)_x_, x = 3, 4, n. Z. Naturforsch. B.

[47] Pérez J., García L., Kessler M., Nolsøe K., Pérez E., Serrano J.L., Martínez J.F., Carrascosa R. (2005). Solid state conformational classification of eight-membered rings in copper complexes double bridged by phosphate, phosphonate or phosphinate ligands. Inorg. Chim. Acta.

[48] Beckmann J., Dakternieks D., Lim A.E.K., Lim K.F., Jurkschat K. (2006). Understanding ring strain and ring flexibility in six- and eight-membered cyclic organometallic group 14 oxides. J. Mol. Struct. THEOCHEM.

[49] Zürcher S., Gramlich V., Togni A. (1999). Germanium-containing ferrocenes and ferrocenophanes. Potential precursors for ring-opening polymerizations and ‘germaferrocenes’. Inorg. Chim. Acta.

[50] Glidewell C., Liles D.C. (1979). The crystal and molecular structure of oxo-bis[tribenzylgermanium(IV)]. J. Organomet. Chem..

[51] Lazraq M., Couret C., Escudie J., Satge J., Draeger M. (1991). Two germaoxetanes from a germene: Formation and structure. Organometallics.

[52] Schilling B., Kaufmann D.E., Kaiser V. (1997). Synthesis and Structure of 2,2′ -Boryl-, Germyl-Silyl-, and Stannyl-Substituted 1,1′-Binaphthyl Systems. Chem. Ber..

[53] Kuz’mina L.G., Struchkov Y.T. (1973). Crystal structure of bis(triphenylgermyl)ether. Russ. J. Struct. Chem..

[54] Glidewell C., Liles D.C. (1978). The crystal and molecular structure of oxobis[triphenylgermanium(IV)]. Acta Crystallogr. Sect. B.

[55] Cui C., Olmstead M.M., Fettinger J.C., Spikes G.H., Power P.P. (2005). Reactions of the Heavier Group 14 Element Alkyne Analogues Ar‘EEAr‘ (Ar‘ = C_6_H_3_-2,6(C_6_H_3_-2,6-Pr*i*_2_)_2_; E = Ge, Sn) with Unsaturated Molecules:  Probing the Character of the EE Multiple Bonds. J. Am. Chem. Soc..

[56] Iwamoto T., Masuda H., Ishida S., Kabuto C., Kira M. (2004). Diverse reactions of nitroxide-radical adducts of silylene, germylene, and stannylene. J. Organomet. Chem..

[57] Sasamori T., Miyamoto H., Sakai H., Furukawa Y., Tokitoh N. (2012). 1,2-Bis(ferrocenyl)digermene: A d−π Electron System Containing a Ge=Ge Unit. Organometallics.

[58] Puff H., Franken S., Schuh W., Schwab W. (1983). Struktur des trimeren Di-t-butylgermaniumoxids. J. Organomet. Chem..

[59] Gurkova S.N., Gusev A.I., Alekseev N.V., Gar T.K., Khromova N.Y. (1985). Crystal and molecular structure of 1,3,5-trigermatris(spiro-1-germa-3,5-disila-3,3,5,5-tetramethyl-4-oxacyclohexa)-2,4,6-cyclotrioxane. Russ. J. Struct. Chem..

[60] Ogawa K., Tanaka K., Yoshimura S., Takeuchi Y., Chien J., Kai Y., Kasai N. (1991). Structure of tetraspiro[1,3,5,7-tetraoxa-2,4,6,8-tetragermacyclooctane-2,1′:4,1′′:6,1′′′:8,1′′′′-tetrakisgerminane]. Acta Crystallogr. Sect. C.

[61] Puff H., Braun K., Franken S., Kök T.R., Schuh W. (1987). Zur chalkogenolyse von monoorganyl-germanium-trichloriden I. Halogenhaltige verbindungen. J. Organomet. Chem..

[62] Nanjo M., Sasage T., Mochida K. (2003). Synthesis and characterization of alkylgermasesquioxanes. J. Organomet. Chem..

[63] Puff H., Franken S., Schuh W. (1983). Hexa-(*t*-butylgermanium-sesquioxid), ein neuartiges käfigmolekül. J. Organomet. Chem..

[64] Puff H., Braun K., Franken S., Kök T.R., Schuh W. (1988). Zur Chalkogenolyse von Monoorganyl-germanium-trichloriden: II. Sesquioxide. J. Organomet. Chem..

[65] Masato N., Takaomi S., Kunio M. (2002). Synthesis of Hexakis(alkylgermasesquioxane)s from Alkyl(chloro)ethoxygermanes and Their Formation Mechanism. Chem. Lett..

[66] Sala A., Pérez-Botella E., Jordá J.L., Cantín A., Rey F., Valencia S. (2021). ITQ-69: A Germanium-Containing Zeolite and its Synthesis, Structure Determination, and Adsorption Properties. Angew. Chem. Int. Ed..

[67] Zaitsev K.V., Kharcheva A.V., Lam K., Zhanabil Z., Issabayeva G., Oprunenko Y.F., Churakov A.V., Zaitseva G.S., Karlov S.S. (2018). Donor-acceptor molecular oligogermanes: Novel properties and structural aspects. J. Organomet. Chem..

[68] Roewe K.D., Rheingold A.L., Weinert C.S. (2013). A luminescent and dichroic hexagermane. Chem. Commun..

[69] Zaitsev K.V., Tafeenko V.A., Oprunenko Y.F., Kharcheva A.V., Zhanabil Z., Suleimen Y., Lam K., Zaitsev V.B., Zaitseva A.V., Zaitseva G.S. (2017). Molecular Oligogermanes and Related Compounds: Structure, Optical and Semiconductor Properties. Chem. Asian J..

[70] Zaitsev K.V., Lam K., Poleshchuk O.K., Bezzubov S.I., Churakov A.V. (2019). Aryl Germanes as Ligands for Transition Polymetallic Complexes: Synthesis, Structure and Properties. Eur. J. Inorg. Chem..

