# Supplementary material for: Structural Motifs in Aryl Organogermanium Ge-O Derivatives for Material Design
Source: Int J Mol Sci. 2023 Sep 1;24(17):13575. doi: 10.3390/ijms241713575 (PMC10487558; doi:10.3390/ijms241713575)
Supplement: Supplementary file 1 [file ijms-24-13575-s001.zip › ijms-2552001-supplementary.pdf]

SUPPORTING INFORMATION

for

Structural Motifs in Aryl Organogermanium Ge-O Derivatives for Materials Design

Table of Contents

NMR Spectral Data

Figure S1. ^{1}H NMR spectrum of $[(p\text{-}FC_6H_4)_3Ge]_2O$ (**1**) ($CDCl_3$, RT)..S2

Figure S2. ^{13}C NMR spectrum of $[(p\text{-}FC_6H_4)_3Ge]_2O$ (**1**) ($CDCl_3$, RT)..S3

Figure S3. ^{19}F NMR spectrum of $[(p\text{-}FC_6H_4)_3Ge]_2O$ (**1**) ($CDCl_3$, RT)..S4

Figure S4. ^{1}H NMR spectrum of $[(p\text{-}F_3CC_6H_4)_3Ge]_2O$ (**2**) ($CDCl_3$, RT)..S5

Figure S5. ^{13}C NMR spectrum of $[(p\text{-}F_3CC_6H_4)_3Ge]_2O$ (**2**) ($CDCl_3$, RT)..S6

Figure S6. ^{19}F NMR spectrum of $[(p\text{-}F_3CC_6H_4)_3Ge]_2O$ (**2**) ($CDCl_3$, RT)..S7

X-Ray Diffraction Analysis Data

Table S1. The crystallographic data for compounds
$[(p\text{-}FC_6H_4)_3Ge]_2O$ (**1**), $[(p\text{-}F_3CC_6H_4)_3Ge]_2O$ (**2**) (structural type **A**)..S8

Table S2. The crystallographic data for compound
cyclo-$[(p\text{-}F_3CC_6H_4)_2GeO]_4$ (**3**) (structural type **F**)..S9

Table S3. The crystallographic data for compound
$[(p\text{-}F_3CC_6H_4)_3GeO]_4Ge$ (**4**) (structural type **K**)..S10

NMR Spectral Data

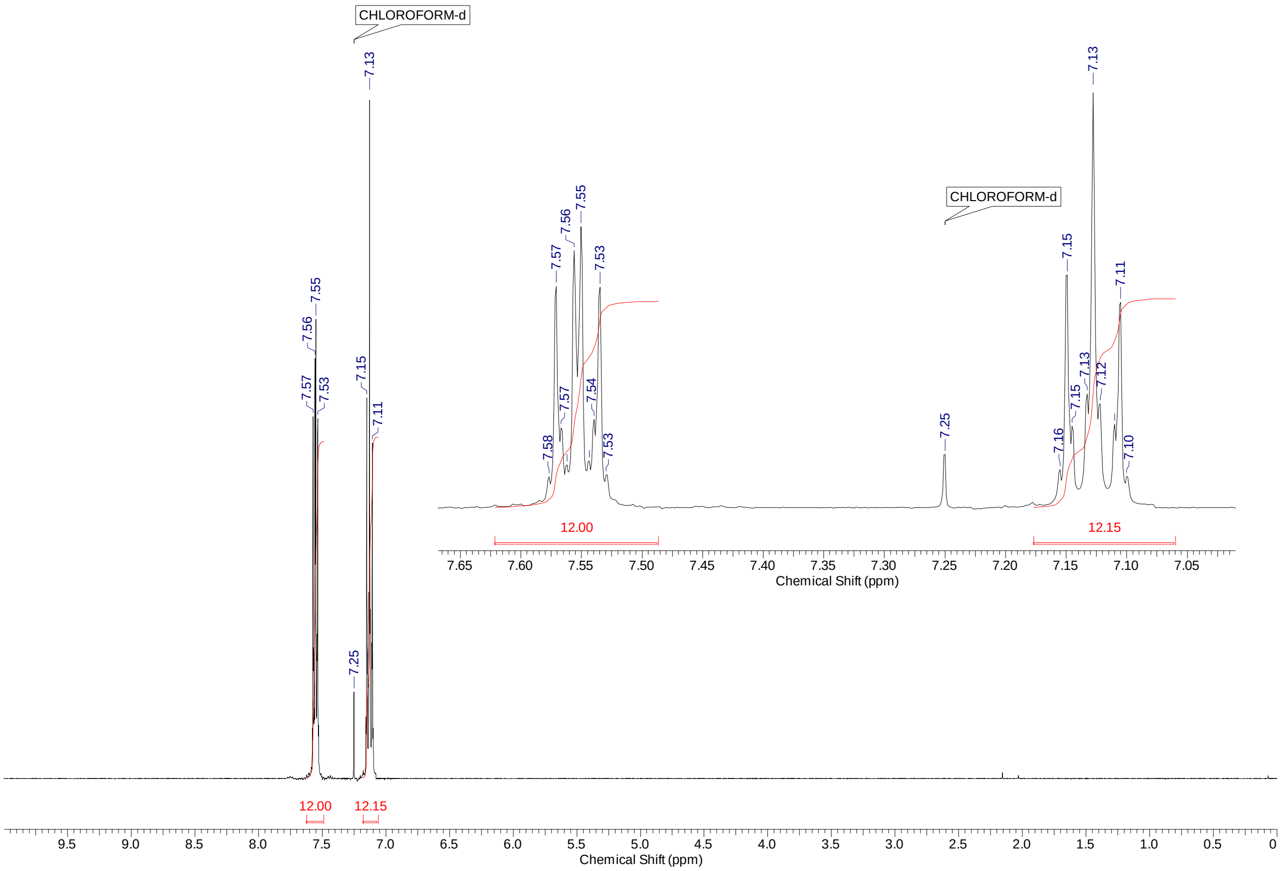

Figure S1. ^{1}H NMR spectrum of $[(p\text{-}FC_6H_4)_3Ge]_2O$ (**1**) ($CDCl_3$, RT).

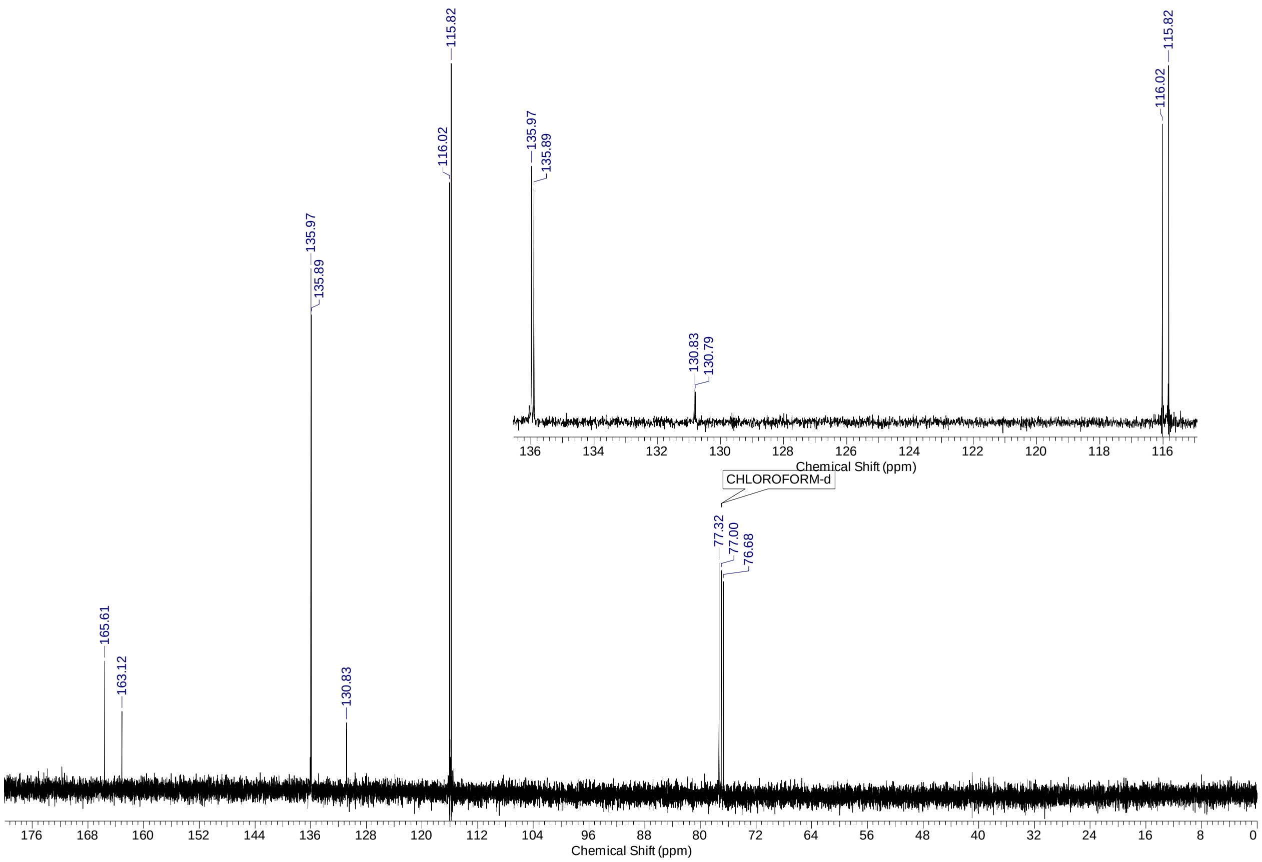

Figure S2. ^{13}C NMR spectrum of $[(p\text{-}FC_6H_4)_3Ge]_2O$ (**1**) ($CDCl_3$, RT).

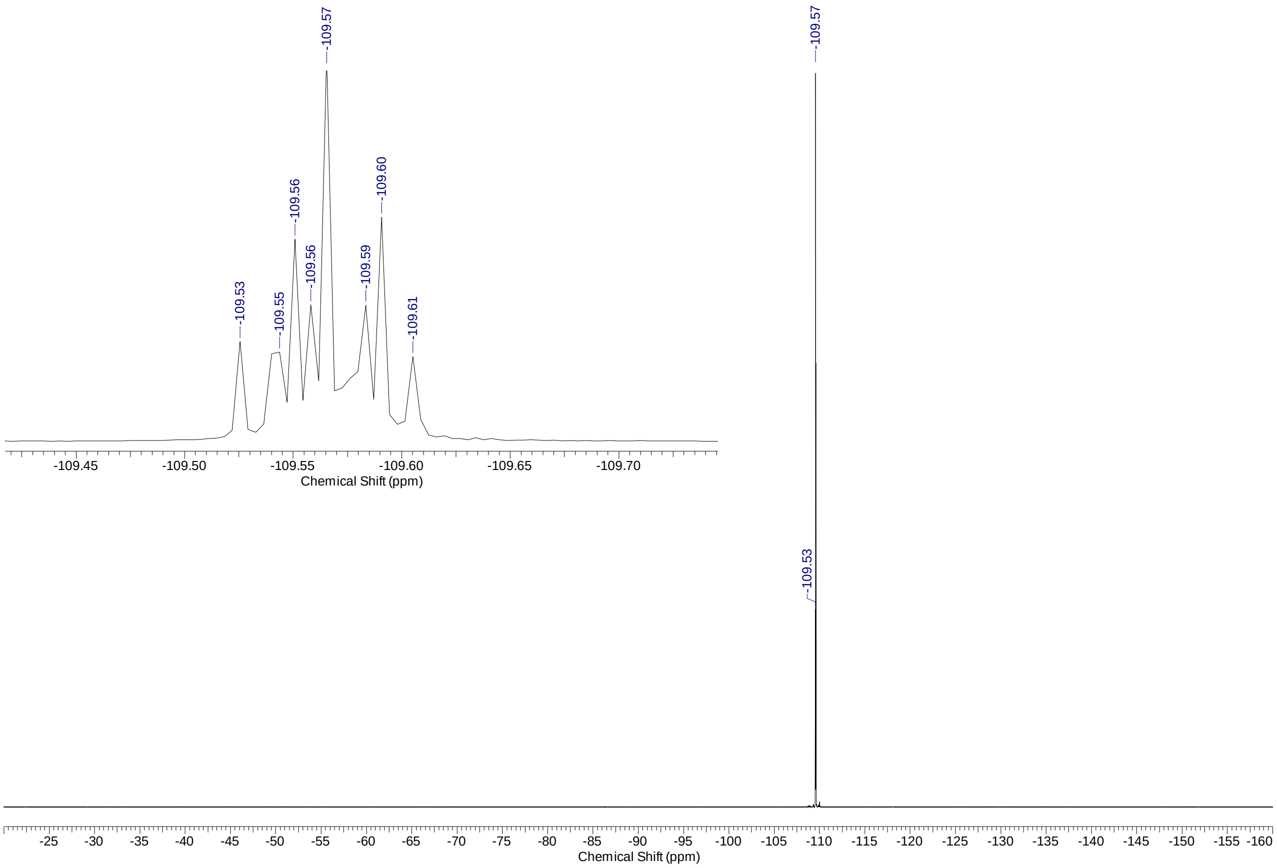

Figure S3. ^{19}F NMR spectrum of [(*p*-$FC_6H_4)_3Ge]_2O$ (**1**) ($CDCl_3$, RT).

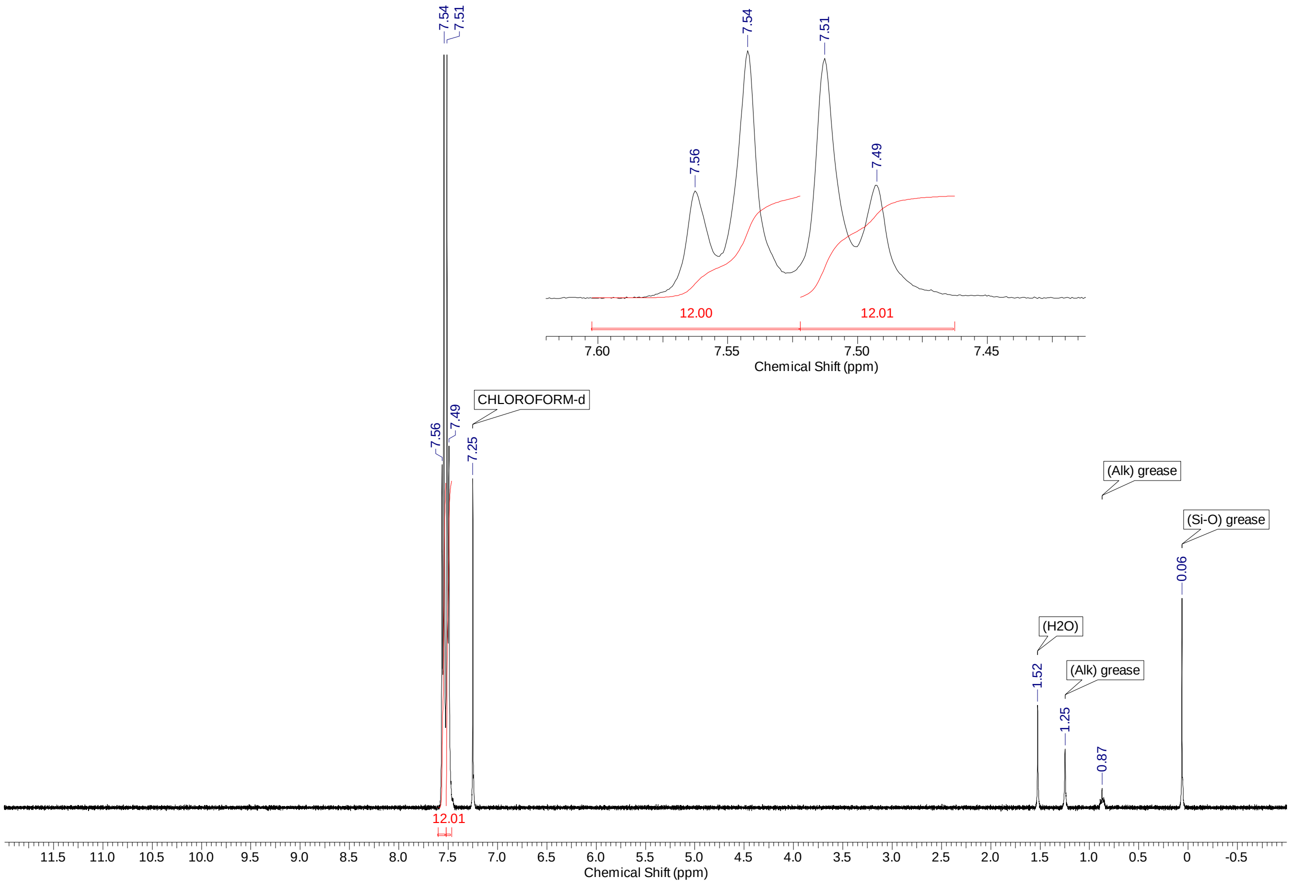

Figure S4. ^{1}H NMR spectrum of $[(p\text{-}F_3CC_6H_4)_3Ge]_2O$ (**2**) ($CDCl_3$, RT).

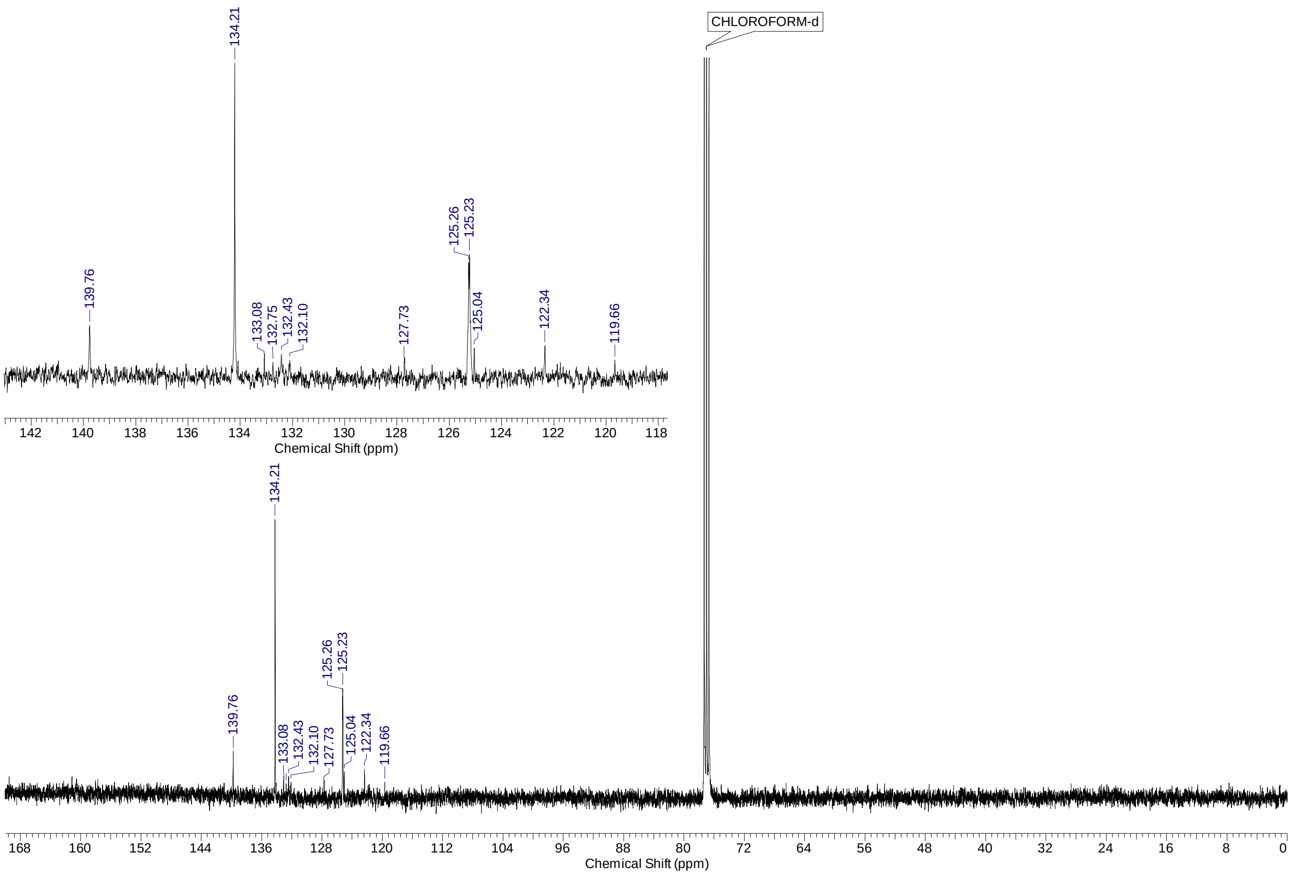

Figure S5. ^{13}C NMR spectrum of $[(p\text{-}F_3CC_6H_4)_3Ge]_2O$ (**2**) ($CDCl_3$, RT).

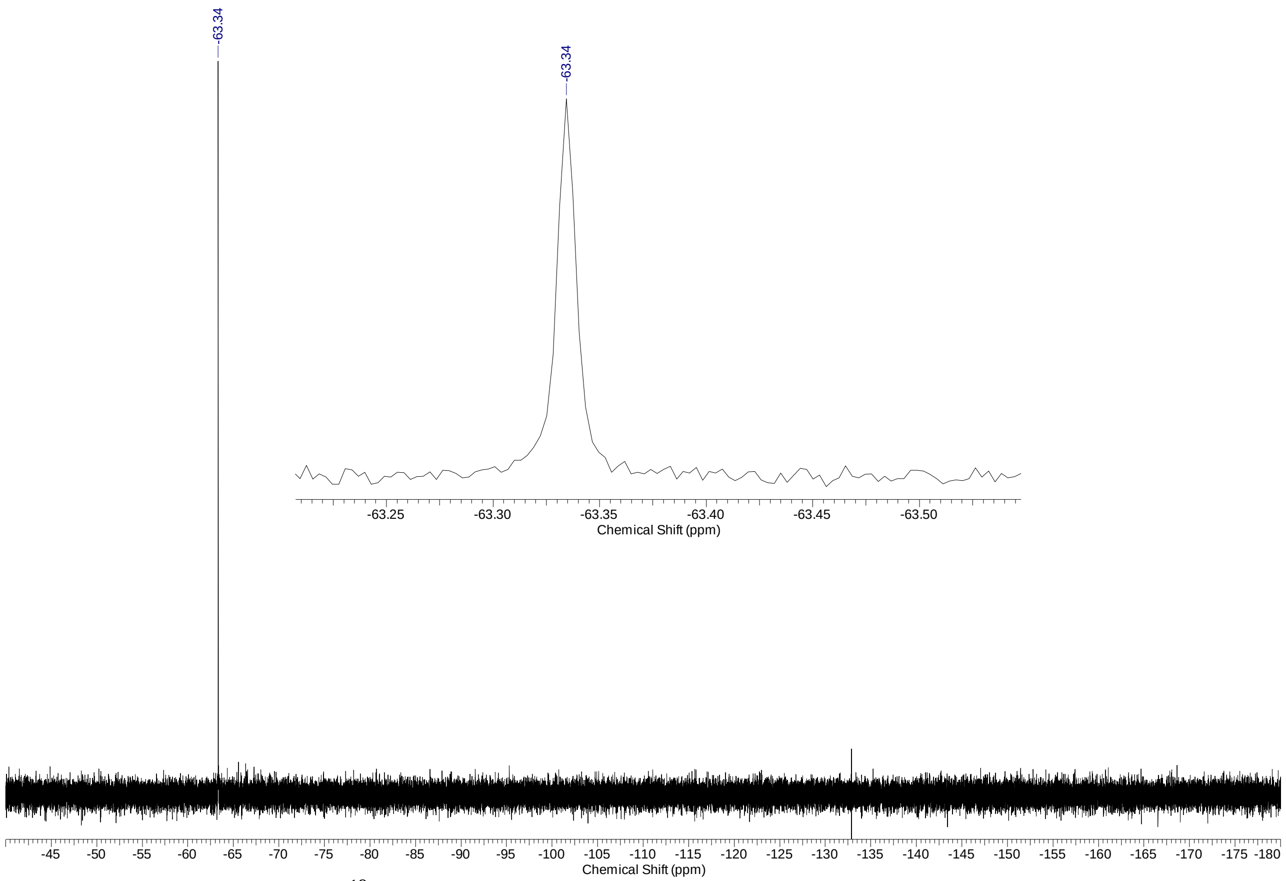

Figure S6. ^{19}F NMR spectrum of $[(p\text{-}F_3CC_6H_4)_3Ge]_2O$ (**2**) ($CDCl_3$, RT).

X-Ray Diffraction Analysis Data

Table S1. The crystallographic data for compounds [(*p*-$FC_6H_4)_3Ge]_2O$ (**1**), [(*p*-$F_3CC_6H_4)_3Ge]_2O$ (**2**) (structural type **A**).

	**1**	**2**
empirical formula	$C_{36}H_{24}F_6Ge_2O$	$C_{42}H_{24}F_{18}Ge_2O$
M_w	731.73	1031.79
temperature (K)	120	150
size (mm)	0.15 x 0.10 x 0.08	0.21 x 0.15 x 0.10
cryst. system	monoclinic	monoclinic
space group	*C*2/c	$P2_1$/c
a (Å)	9.7200(4)	16.4116(13)
b (Å)	20.9864(8)	18.5115(14)
c (Å)	15.2582(6)	13.8675(11)
β (deg)	99.143(1)	107.325(1)
V ($Å^3$)	3072.9(2)	4021.9(5)
Z	4	4
ρ_{cald} ($g*cm^{-3}$)	1.582	1.704
abs coeff. (mm^{-1})	2.022	1.613
F(000)	1464	2040
θ range (deg)	2.33 - 28.00	2.54 - 27.00
no. of collected/unique rflns.	12118 / 3681	31468 / 8762
R_{int}	0.0193	0.0549
data/restraints/params.	3681 / 9 / 272	8762 / 1026 / 838
goodness of fit on F^2	1.074	1.107
final *R* indices ($I > 2\sigma(I)$)	R_1 = 0.0552, wR_2 = 0.1196	R_1 = 0.0412, wR_2 = 0.0925
R indices (all data)	R_1 = 0.0701, wR_2 = 0.1298	R_1 = 0.0637, wR_2 = 0.1020
largest diff. peak/hole (e/$Å^3$)	1.754 / -1.771	0.766 / -0.715

Table S2. The crystallographic data for compound *cyclo*-[(*p*-$F_3CC_6H_4)_2GeO]_4$ (**3**) (structural type **F**).

empirical formula	$C_{56}H_{32}F_{24}Ge_4O_4$
M_w	1515.17
temperature (K)	120
size (mm)	0.30 x 0.20 x 0.20
cryst. system	tetragonal
space group	$I4_1/a$
a (Å)	11.2193(5)
c (Å)	44.188(4)
V (Å^3)	5562.1(7)
Z	4
ρ_{cald} (g*cm^{-3})	1.809
abs coeff. (mm^{-1})	2.269
F(000)	2976
θ range (deg)	2.57 - 27.99
no. of collected/unique rflns.	14852 / 3351
R_{int}	0.0786
data/restraints/params.	3351 / 282 / 234
goodness of fit on F^2	1.144
final *R* indices ($I > 2\sigma(I)$)	R_1 = 0.1030, wR_2 = 0.2147
R indices (all data)	R_1 = 0.1170, wR_2 = 0.2218
largest diff. peak/hole (e/Å^3)	1.002 / -1.015

Table S3. The crystallographic data for compound $[(p\text{-}F_3CC_6H_4)_3GeO]_4Ge$ (**4**) (structural type **K**).

empirical formula	$C_{84}H_{48}F_{36}Ge_5O_4*2(C_6H_6)$
F_w	2324.39
temperature (K)	183
size (mm)	0.25 x 0.25 x 0.20
cryst. system	tetragonal
space group	$P4_32_12$
a (Å)	16.5336(8)
c (Å)	34.892(3)
V (Å^3)	9538.1(9)
Z	4
ρ_{cald} (g*cm^{-3})	1.619
abs coeff. (mm^{-1})	1.679
$F(000)$	4608
θ range (deg)	2.10 - 27.00
no. of collected/unique rflns.	92313 / 10419
R_{int}	0.0416
data/restraints/params.	10419 / 1194 / 894
goodness of fit on F^2	1.108
final R indices ($I > 2\sigma(I)$)	$R_1 = 0.0445$, w$R_2 = 0.1199$
R indices (all data)	$R_1 = 0.0528$, w$R_2 = 0.1199$
largest diff. peak/hole (e/Å^3)	0.809 / -0.561
Flack param.	0.001(3)
